# *Phyllocnistis furcata* sp. nov.: a new species of leaf-miner associated with *Baccharis* (Asteraceae) from Southern Peru (Lepidoptera, Gracillariidae)

**DOI:** 10.3897/zookeys.996.53958

**Published:** 2020-11-24

**Authors:** José Cerdeña, Jackie Farfán, Héctor A. Vargas, Rosângela Brito, Gislene L. Gonçalves, Ana Lazo, Gilson R. P. Moreira

**Affiliations:** 1 PPG Biologia Animal, Departamento de Zoologia, Instituto de Biociências, Universidade Federal do Rio Grande do Sul, Av. Bento Gonçalves, 9500, Porto Alegre, RS 91501-970, Brazil Universidad Nacional de San Agustín de Arequipa Arequipa Peru; 2 Universidad Nacional de San Agustín de Arequipa, Museo de Historia Natural, Av. Alcides Carrión s/n, Arequipa, Peru Universidade Federal do Rio Grande do Sul Porto Alegre Brazil; 3 Departamento de Recursos Ambientales, Facultad de Ciencias Agronómicas, Universidad de Tarapacá, Casilla 6-D, Arica, Chile Universidad de Tarapacá Arica Chile; 4 Embrapa Cerrados, Planaltina, Distrito Federal, Brazil Universidad Nacional de San Agustín de Arequipa Arequipa Peru; 5 Departamento de Zoologia, Instituto de Biociências, Universidade Federal do Rio Grande do Sul, Av. Bento Gonçalves 9500, Porto Alegre RS, 91501-970, Brazil Universidade Federal do Rio Grande do Sul Porto Alegre Brazil; 6 Universidad Nacional de San Agustín de Arequipa, Laboratorio Fisiologia Animal, Av. Alcides Carrión s/n, Arequipa, Peru Universidad de Tarapacá Arica Chile

**Keywords:** Andes, Arequipa, barcoding, immature stages, Phyllocnistinae

## Abstract

The southwestern Andes of Peru harbors a hidden taxonomic diversity of Lepidoptera. Here a new leaf-mining species of Gracillariidae (Lepidoptera) is described, *Phyllocnistis furcata* Vargas & Cerdeña, **sp. nov.**, from a dry Andean valley of southern Peru, at 2400 m above sea level. The morphological aspects of adults (male and female) and the immature stages associated with *Baccharis alnifolia* Meyen & Walp. (Asteraceae) are given, under optical microscopy and scanning electron microscopy. DNA barcodes show that its nearest neighbor is the Atlantic Forest species *Phyllocnistis ourea* Brito & Moreira, 2017 that feeds on *Baccharis anomala* DC. The importance of morphological characters from immature stages for diagnosis among congeneric species is also discussed. *Phyllocnistis furcata* represents the fourth species of *Phyllocnistis* Zeller for Peru, and first record from the south of Peru for the genus.

## Introduction

The Andes region of Peru contains hotspots of biodiversity for plants and animals (Myers et al. 2000). It includes global record highs of species richness and endemism rates for many taxa of Lepidoptera, particularly along the eastern slopes of the Andes (Lamas 2003; Pyrcz 2004; Hall 2005; Ignatov et al. 2011; Willmott et al. 2011; Pyrcz et al. 2014; Sublett et al. 2019). On the other hand, the level of knowledge of the lepidopteran fauna on the southwestern slopes of the Peruvian Andes is poor, based on a small number of studies of butterflies (Lamas 1977; Cerdeña et al. 2014; Farfán 2018; Farfán et al. 2020b), and with recent records of moths (Cerdeña et al. 2019; Farfán et al. 2020a), including the first record of a Gracillariidae species for this region (Davis et al. 2020).

Gracillariidae represents one of the most diverse families of micromoths with 1995 recognized species distributed in more than 100 genera (van Nieukerken et al. 2011; De Prins and De Prins 2020). Larvae are predominantly leaf-miners although some species mine stems or fruits (Guillén et al. 2001), and others bore into flowers (Vargas and Landry 2005), fruits (Hu et al. 2011) or stems (Davis et al. 1991), and may also be leaf-rollers or gall inducers (Hanson et al. 2014; Kawakita and Kato 2016; Vargas-Ortiz et al. 2019). In the Neotropics, more intense taxonomic work has been performed in the last decade, resulting in 28 newly described species (De Prins and De Prins 2020). Despite this effort of documenting the fauna, currently numbering 204 Neotropical species, there are gaps in information for gracillariids in several biodiversity hotspots, such as the Andes. Particularly in Peru, currently only 28 species of gracillariids are known (Kawakita et al. 2019; Davis et al. 2020; De Prins and De Prins 2020). From these, one is a non-native species introduced in the coastal area (Castillo and Cornejo 1996), two species were recently reported from southwestern Peru (Vargas 2010; Davis et al. 2020), five were described from northern Peru (Kawakita et al. 2019), and 19 species were described in the early last century by Edward Meyrick (Meyrick 1915, 1921) from material collected by Herbert Simpson Parish from the central Peruvian coast (Lima), central Andes (Matucana, Oroya, Huancayo, Jauja), and northeastern Peruvian Amazon (Iquitos, Yurimaguas) during two collecting trips to the tropics in 1914 and 1920 (Alexander 1916, 1921). These species in particular remain only known from the type specimens, some of which have deteriorated (Brito et al. 2017; De Prins et al. 2019).

*Phyllocnistis* Zeller, 1848 is a genus of Gracillariidae with 112 named species distributed in all biogeographic regions except Antarctica (Brito et al. 2016, 2017; Fochezato et al. 2018; Kirichenko et al. 2018; De Prins and De Prins 2020). A total of 28 species has been reported for the Neotropical region (De Prins et al. 2019), with 16 species recorded in the last ten years (Kawahara et al. 2009; Davis and Wagner 2011; Brito et al. 2012, 2017, 2019; Fochezato et al. 2018). However, only three species were registered from Peru: *Phyllocnistis sciophanta* Meyrick, 1915, *P. sexangula* Meyrick, 1915, and *P. citrella* Stainton, 1856; the first two species, collected from the center of Peru (Department of Lima) more than 100 years ago, remain with their host plant and immature stages unknown (Brito et al. 2017; De Prins et al. 2019). The third species, with a worldwide distribution, known to be a pest in citrus fruits, is a native from Asia (Castillo and Cornejo 1996; De Prins et al. 2019). This lack of data is mainly due to two conditions that prevailed for a long time, not only in Peru but also in other countries of the Neotropical region (Brito et al. 2016): low collection intensity and scarce taxonomy activity on Gracillariidae and Microlepidoptera in general.

The hypermetamorphic development of the larvae of *Phyllocnistis* typically comprises two endophytic forms (e.g., Fochezato et al. 2018; Brito et al. 2019). The early, sap-feeding larva actively mines specific host tissues; later, the spinning larva does not feed and has most of the buccal apparatus atrophied, but has a functional spinneret that is used to expel silk to construct the pupal cocoon.

A variety of host plants are associated with *Phyllocnistis* in the Neotropical region, including 15 genera from 13 different plant families (Brito et al. 2017). Three species are known to be associated with the genus *Baccharis* (Asteraceae): *P. baccharidis* Hering, 1958, *P. ourea* Brito & Moreira, 2017, and an undescribed species associated with *Baccharis trimera* (Brito et al. 2017), the first from Argentina and other two from Brazil. This Neotropical plant genus is characterized by the tufted indumentum of leaves and stems, and by the unisexual florets generally in separate specimens (Müller 2006), currently comprising 440 species (Heiden et al. 2019).

Recently, as part of an ongoing study on the diversity of microlepidopterans in the Andes in southern Peru, we found a leaf-mining species of *Phyllocnistis* associated with *Baccharis*. Comparison at both morphological and molecular levels showed that it does not conform to any known *Phyllocnistis* species. The morphological description of adults (male and female) and immature stages of this new species is herein given. We also present a preliminary analysis of mitochondrial (COI) DNA sequences including congeneric Neotropical species.

## Materials and methods

Larvae and pupae found in mines on leaves of *Baccharis alnifolia* Meyen & Walp. (Asteraceae) in the locality of Characato (16°27'S, 71°28'W), 2400 m, Characato Municipality, Arequipa Department, Peru, were collected and reared in plastic cups, at constant abiotic conditions (20 ± 2 °C, 13:11 h photoperiod) in the laboratory of Area de Entomología, Museo de Historia Natural, Universidad Nacional de San Agustin, Arequipa city, Peru, during September 2018, April 2019, and November 2019.

In total, 58 specimens have been studied: 23 adults, 17 larvae, 20 pupae. Adults that emerged from the mines were pinned and dried, and immature stages were fixed with Dietrich´s fluid and preserved in 70% ethanol. Genitalia were cleared by heating in hot 10% KOH for ~ 15 minutes. They were subsequently stained with Chlorazol black and Eosin, and then slide-mounted with Euparal.

Morphological observations were performed with the aid of a Zeiss Stemi305, and structures selected to be illustrated were photographed with a Nikon SMZ25 stereomicroscope. Vectorized line drawings were then made with the software CorelDraw X4, using the corresponding digitalized images as a guide. The terminology used for descriptions of adult wing pattern, genitalia and immature stages follows Brito et al. (2017, 2019).

For scanning electron microscope analyses, specimens were dehydrated in a Bal-tec CPD030 critical-point dryer, mounted with double-sided tape on metal stubs, and coated with gold in a Bal-tec SCD050 sputter coater. They were then examined and photographed in a JEOL JSM6060 scanning electron microscope at the Centro de Microscopia Eletrônica (CME) of Federal University of Rio Grande do Sul (UFRGS).

For plant anatomical descriptions, field-collected leaf portions (approx. 0.3 cm^2^) of *B. alnifolia* containing mines of *P. furcata* were fixed in FAA (37% formaldehyde, glacial acetic acid, and 50% ethanol, 1:1:18, v/v) for 24 h. They were then dehydrated in a series of ethanol (40%, 70%, 90%, 96%); embedded in paraffin and sectioned transversely (7 µm) on a rotary microtome. The sections were adhered to a microscope slide glass, then observed and photographed without staining, by using a Nikon SMZ25 stereomicroscope.

Total genomic DNA was extracted from larval tissue (last sap-feeding instar) of five specimens (H86–H90), using the CTAB method (Doyle and Doyle 1987), to support the hypothesis that the morphologically distinct specimens studied confirm a new *Phyllocnistis* species, and explore the phylogenetic placement among Neotropical congeners. We amplified part of the mitochondrial gene cytochrome oxidase I (COI – 639 bp) using primers and conditions described by Folmer et al. (1994). PCR products were purified using Exonuclease I (GE Healthcare Inc.) and Shrimp Alkaline Phosphatase (SAP), sequenced with forward and reverse primers using a BigDye kit, and analyzed on an ABI3730XL (Applied Biosystems Inc.). Chromatograms obtained from the automatic sequencer were read and sequences were assembled using the software CodonCode Aligner (CodonCode Corporation). The new sequences obtained in this study are publicly available in GenBank and BOLD (DS-GRANEO) databases (Table 1). To explore the phylogenetic position of the new taxon and its specific classification we combined our COI data with a published dataset of ten species and 13 undescribed lineages of Neotropical *Phyllocnistis* (Davis and Wagner 2011; Brito et al. 2012, 2017, 2019; Lees et al. 2013) (Table 1). This includes *P. ourea* that feeds on *Baccharis anomala*, and *Phyllocnistis* sp. 12 (Brito et al. 2017) associated to *Baccharis trimera* (Less) DC. *Angelabella tecomae* Vargas & Parra, 2005 (Oecophyllembiinae) and *Marmara arbutiella* Busck, [1904] (Marmarinae) were used as outgroups as they represent subfamilies closely related to Phyllocnistinae (Kawahara et al. 2017). A distance tree based on Neighbor-joining (NJ) method was generated from 31 nucleotide sequences using Kimura 2-parameters (K2P) model in MEGA X (Kumar et al. 2018). The evolutionary history was inferred by using the Maximum Parsimony (MP) analysis using the Tree-Bisection-Regrafting (TBR) algorithm with search level 1, with initial trees obtained by random addition of sequences (10 replicates) also in MEGA X. A Maximum Likelihood (ML) analysis was also performed, with the substitution model GTR+G+I according to the Akaike Information Criterion (AIC) estimated by JMODELTEST (Posada 2008), using PHYML 3.0 (Guindon et al. 2010). Initial trees for the heuristic search were obtained automatically with BioNJ algorithm to a matrix of pairwise distances. Monophyly-confidence limits of all analysis were assessed with the bootstrap (BS) method after 1000 bootstrap iterations. Sequence divergences were quantified using K2P model for (i) the genus *Phyllocnistis* (using 35 named species deposited in BOLD; Table 1), (ii) the Neotropical *Phyllocnistis* (10 described + 13 undescribed species, Table 1), and (iii) the new species vs. *Baccharis*-feeding lineages.

**Table 1.** Specimens used for molecular analyses of *Phyllocnistis furcata* sp. nov. Both the Sample ID and Process ID codes are unique identifiers linking the record in the BOLD database and the voucher specimen from which the sequence is derived. The asterisk(s) indicates those specimens associated with the *Baccharis* as host plant: *B. alnifolia, **B. anomala, ***B. trimera.

Species	Sample ID	Process ID	GenBank accession	Reference
*Phyllocnistis furcata* sp. nov.*	H86	MISA051-20	MT832361	This study
*Phyllocnistis furcata* sp. nov.*	H87	MISA052-20	MT832362	This study
*Phyllocnistis furcata* sp. nov.*	H88	MISA053-20	MT832363	This study
*Phyllocnistis furcata* sp. nov.*	H89	MISA054-20	MT832364	This study
*Phyllocnistis furcata* sp. nov.*	H90	MISA055-20	MT832365	This study
*Phyllocnistis hemera*	LMCI 292-25C	MISA019-17	MG264519	Fochezato et al. 2018
*Phyllocnistis kawakitai*	AK0105	GRANO105-11	KF460801	Lees et al. 2014
*Phyllocnistis norak*	CLV1381	LNOUC318-10	JN276191	Lees et al. 2014
*Phyllocnistis ohshimai*	CLV1367	LNOUC304-10	JN276189	Lees et al. 2014
Phyllocnistis ourea**	LMCI 297-15B	MISA013-16	KY006927	Brito et al. 2016
*Phyllocnistis petronellii*	IO0536	LEPPC2394-16	KY682706	Brito et al. 2017
*Phyllocnistis perseafolia*	DDAV-D555	RDOPO393-10	HM382096	Davis and Wagner 2011
*Phyllocnistis phoebus*	LMCI 263-9	MISA014-16	KY006929	Brito et al. 2016
*Phyllocnistis selene*	LMCI 263-22	MISA015-16	KY006928	Brito et al. 2016
*Phyllocnistis tethys*	LMCI 174-55-1	GBMIN15477-13	JX272049	Brito et al. 2012
*Phyllocnistis* sp. 2	AK0198	LNOUD2290-12	KF460914	Lees et al. 2014
*Phyllocnistis* sp. 3	AK0210	LNOUD2302-12	KF460586	Lees et al. 2014
*Phyllocnistis* sp. 4	AYK-FG10-135	LNOUC1229-11	KF460667	Lees et al. 2014
*Phyllocnistis* sp. 5	CLV1284	LNOUD1191-12	KF460613	Lees et al. 2014
*Phyllocnistis* sp. 7	CLV1368	LNOUC305-10	JN276190	Lees et al. 2014
*Phyllocnistis* sp. 9	CLV2993	LNOUD336-11	KF460927	Lees et al. 2014
*Phyllocnistis* sp. 10	CLV3313	LNOUD489-11	KF460904	Lees et al. 2014
*Phyllocnistis* sp. 11	CLV4347	LNOUD776-12	KF460865	Lees et al. 2014
*Phyllocnistis* sp. 12***	CLV5900 and CLV5901	GRPAL1220-13 and GRPAL1221-13	KY682713 and KF460659	Lees et al. 2014
*Phyllocnistis* sp. 13	CLV5902	GRPAL1222-13	KY682713	Lees et al. 2014
*Phyllocnistis* sp. 15	LEAFMINE2015-0006	LEPPC1378-15	KY682712	Brito et al. 2017
*Phyllocnistis* sp. 16	LEAFMINE2015-0008	LEPPC1380-15	KY682711	Brito et al. 2017
*Phyllocnistis* sp. 17	LEAFMINE2015-0010	LEPPC1382-15	KY682704	Brito et al. 2017

Abbreviations for the museum collections and institutions from which specimens were examined are:

**LMCI** Laboratório de Morfologia e Comportamento de Insetos, Universidade Federal do Rio Grande do Sul, Porto Alegre, Rio Grande do Sul, Brazil.

**MUSA**Museo de Historia Natural, Universidad Nacional de San Agustín de Arequipa, Arequipa, Perú.

**MUSM** Museo de Historia Natural, Universidad Nacional Mayor San Marcos, Lima, Peru.

## Results

### Phyllocnistis furcata

Taxon classificationAnimaliaLepidopteraGracillariidae

Vargas & Cerdeña sp. nov.

3D27D75A-A5C9-5B17-8E17-0D6C33EE0F7F

http://zoobank.org/F54378EF-7ADF-425D-9FDD-B8F2223D1381

**Figure 1.**
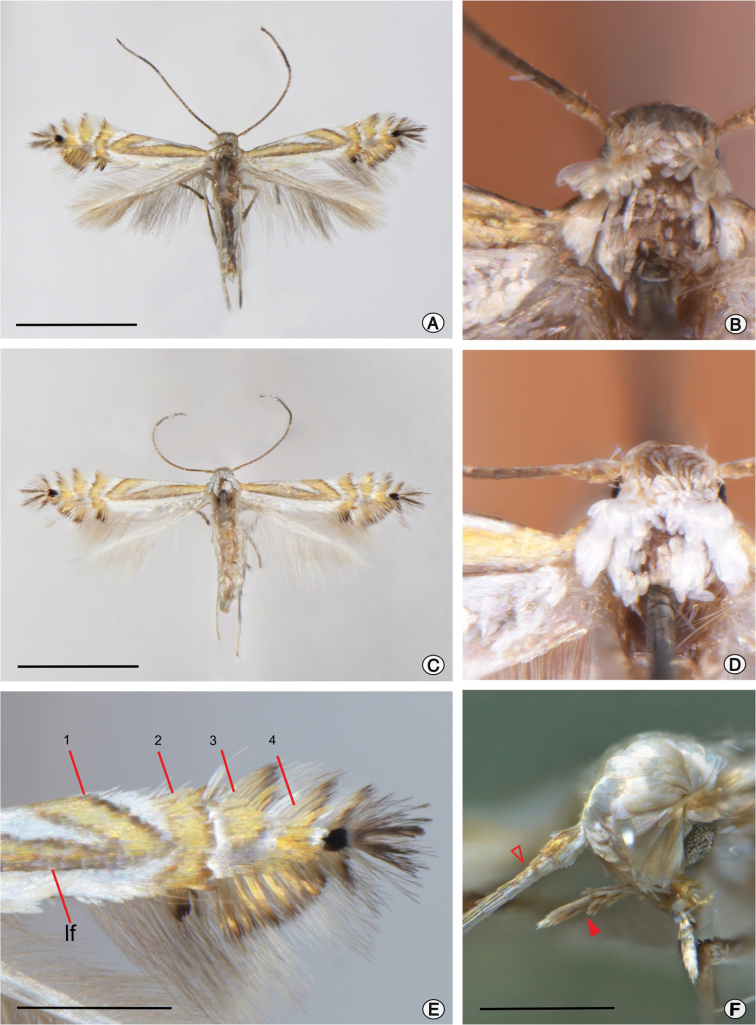
*Phyllocnistis furcata* sp. nov. adult morphology **A, B** holotype of *P. furcata*, male, with head in detail, dorsal view (MUSM) **C, D** paratype of *P. furcata*, female, with head in detail, dorsal (MUSA_ENT 015143) **E** detail of right forewing with terminologies adopted, lf: longitudinal fascia; tf (1–4) transverse fascia(e) **F** lateral view of a male head with labial palpus (indicated by closed arrow head) and antenna (indicated by open arrow head). Scale bars: 2 mm (**A, C**), 1 mm (**E**), 0.4 mm (**F**).

**Figure 2.**
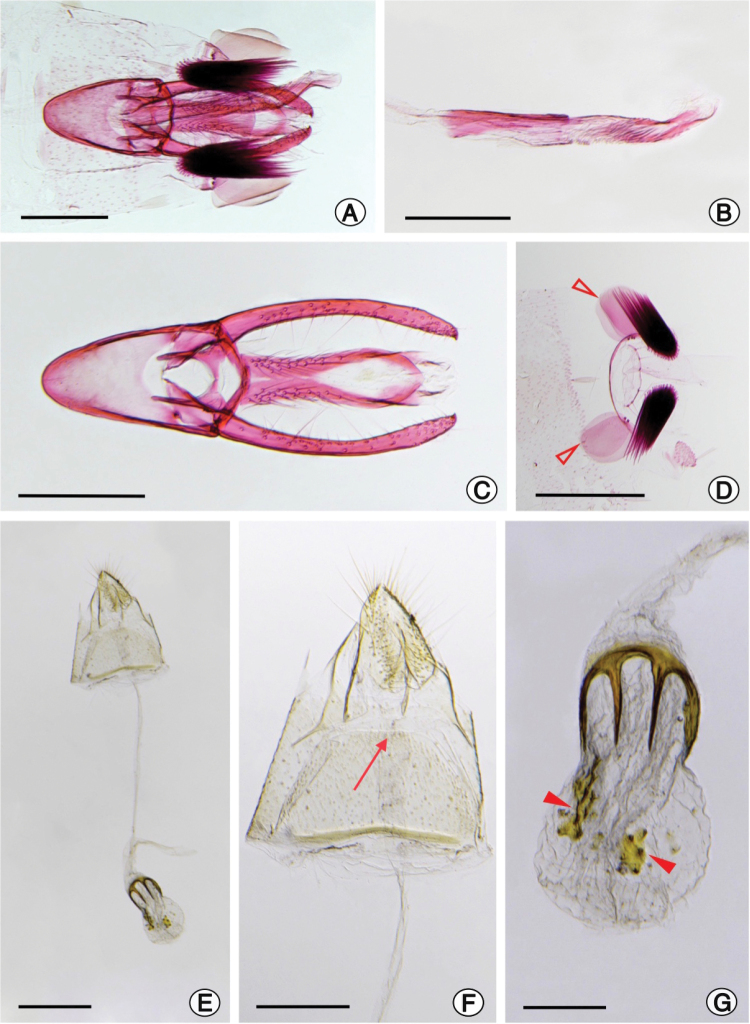
*Phyllocnistis furcata* sp. nov. genital morphology under light microscopy **A** male genital segments, ventral view **B** phallus, lateral **C** male genitalia with phallus removed, ventral **D** coremata (open arrows indicate wide rounded flat scales) **E** female genitalia, ventral **F** female last abdominal segments, in detail (seta points to ostium bursae) **G** corpus bursae in detail (closed arrows indicate two small signa with minute dark spots). Scale bars: 0.2 mm (**A–C, F, G**), 0.3 mm (**D**), 0.4 mm (**E**). Genitalia slides: MUSA_Gent_015142 (A), MUSA_Gent_015146 (**B–D**), MUSA_Gent_015148 (**E–G**).

**Figure 3.**
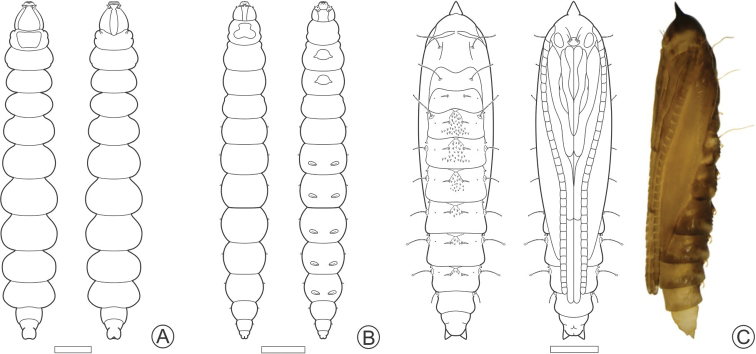
Larval and pupal morphology of *P. furcata* sp. nov. under light microscopy **A** sap-feeding larva, dorsal and ventral view **B** spinning larva, dorsal and ventral **C** pupa, dorsal, ventral, and lateral, respectively. Scale bars: 500 µm.

**Figure 4.**
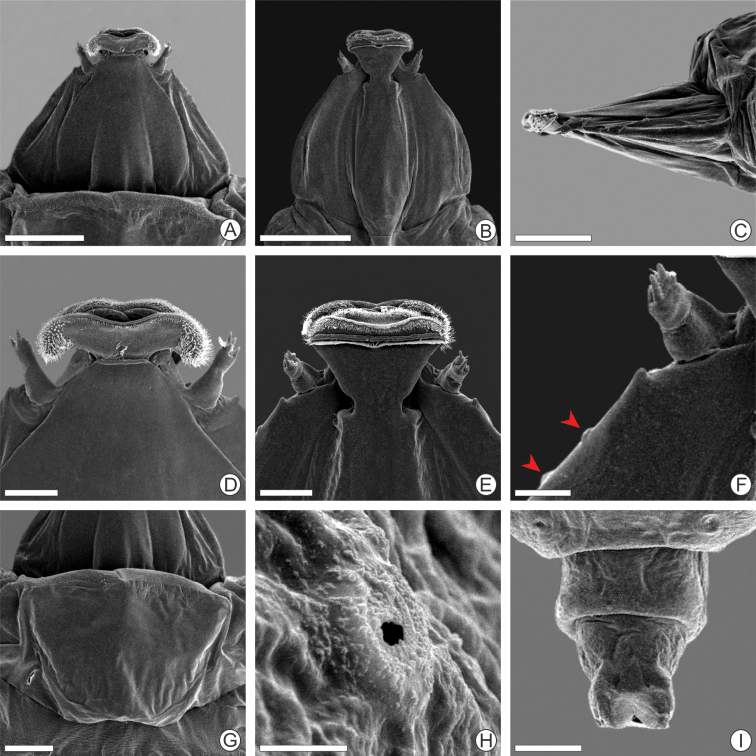
Scanning electron micrographs of *P. furcata* sp. nov. sap-feeding larva **A–C** head under dorsal, ventral, and lateral views **D** labrum, dorsal **E** labium, ventral **F** antenna and stemmata (indicated by arrow), ventral **G** prothoracic shield, dorsal **H** abdominal spiracle, lateral **I** last abdominal segment, ventral. Scale bars: 200 µm (**A, B**), 150 µm (**C**), 50 µm (**D, E**), 25 µm (**F**), 100 µm (**G, I**), 5 µm (**H**).

**Figure 5.**
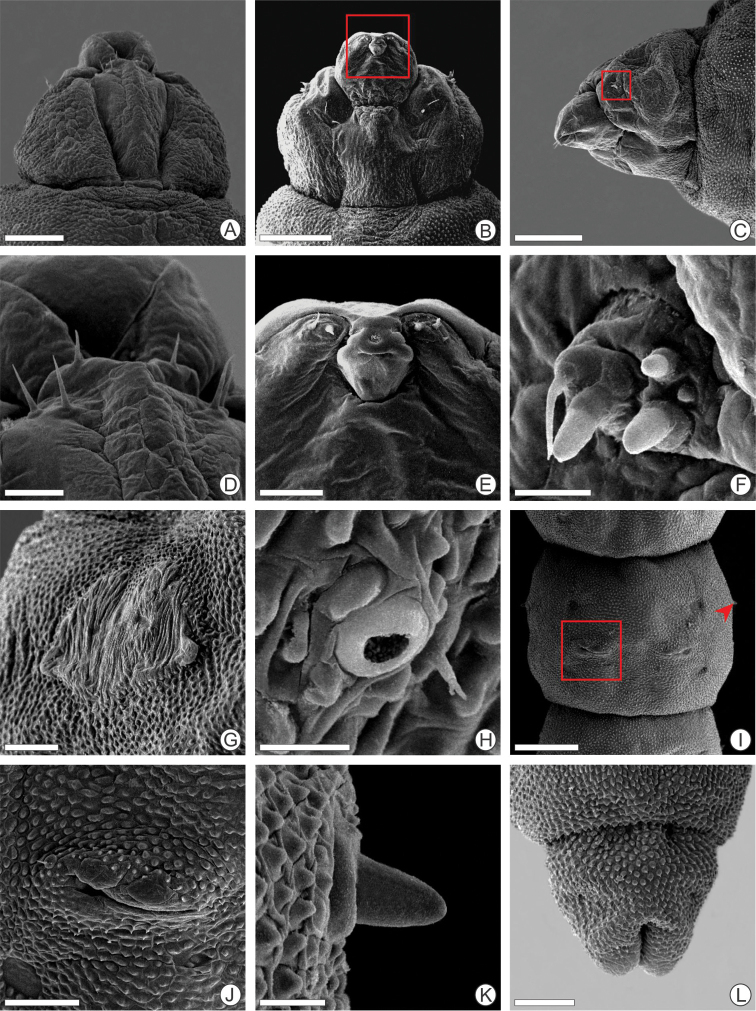
Scanning electron micrographs of *P. furcata* sp. nov. spinning larva **A–C** head under dorsal, ventral, and lateral views respectively **D** detail of trophic lobe and clypeal region, dorsal **E** spinneret, (indicated by square in B) **F** antenna, lateral (indicated by square in C) **G** mesothoracic ambulatory callus in detail, ventral **H** abdominal spiracle, lateral **I** abdominal segment A6, ventral (campaniform sensilla indicated by arrow) **J** abdominal ambulatory callus in detail, ventral (indicated by square in I) **K** campaniform sensilla in detail, ventral (indicated by arrow in I) **L** last abdominal segment, dorsal. Scale bars: 100 µm (**A, B**), 150 µm (**C, I**), 25 µm (**D, E**), 10 µm (**F, H, K**), 50 µm (**G, L**), 40 µm (**J**).

**Figure 6.**
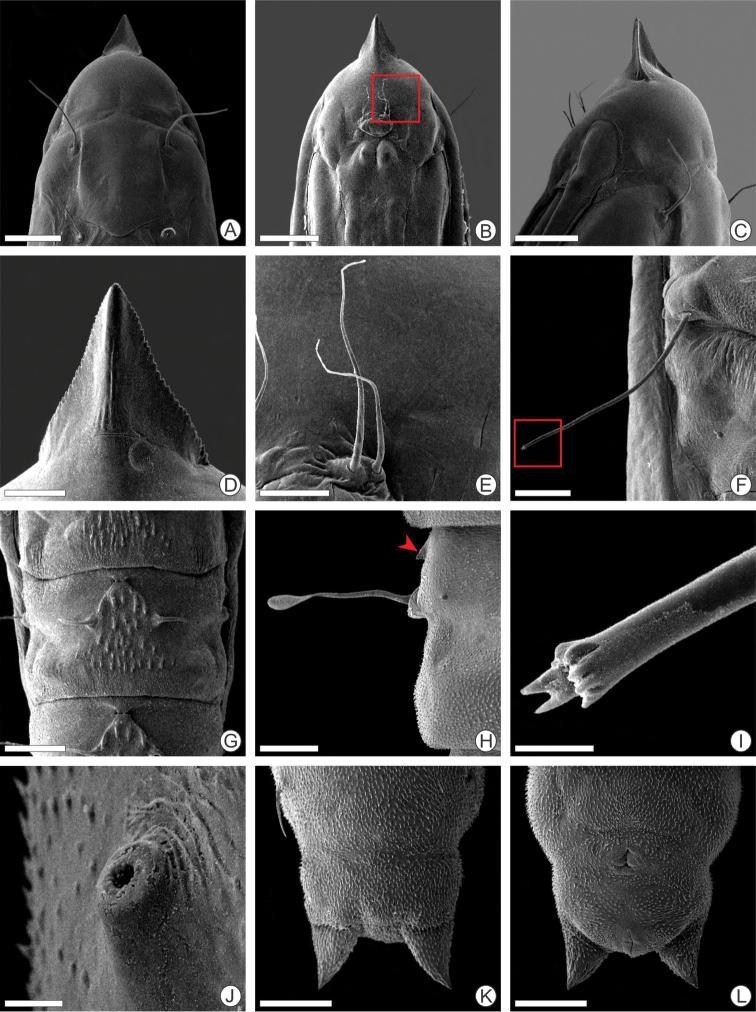
Scanning electron micrographs of *P. furcata* sp. nov. pupa **A–C** head under dorsal, ventral, and lateral views **D** cocoon-cutter, ventral **E** setae on clypeus, ventral (indicated by square in B) **F** lateral seta on abdominal segment A2, dorsal **G** detail of abdominal segment A3, dorsal **H** lateral seta with clavate apex, adjacent to spiracle (indicated by arrow) and close to small spine on abdominal segment A6, dorsal **I** detail of lateral seta distal portion, with dentate apex from abdominal segment A2, (indicated by square in F) **J** abdominal spiracle, lateral **K, L** last abdominal segments, dorsal and ventral, respectively. Scale bars: 200 µm (**A–C, G**), 50 µm (**D**), 40 µm (**E**), 100 µm (**F, H, K, L**), 10 µm (**I, J**).

**Figure 7.**
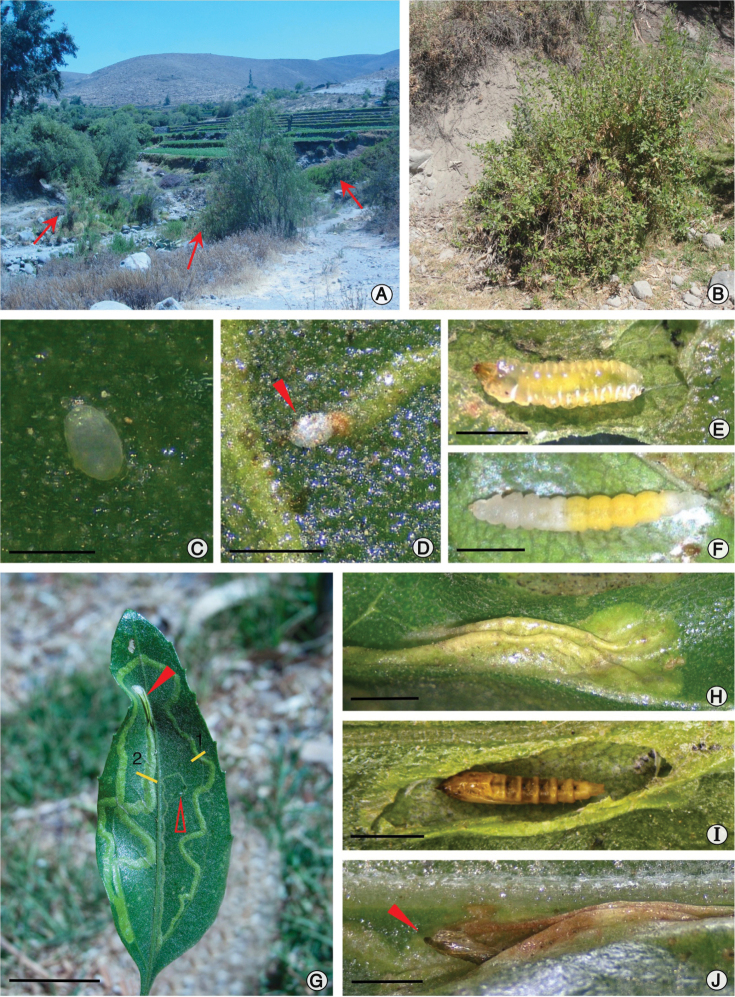
Natural history of *P. furcata* sp. nov. **A** type locality in Characato valley, Arequipa, southern Peru (setae point to *Baccharis* host-plants) **B***B. alnifolia* plant, under close view **C** egg containing developing embryo **D** early mine with attached egg shell remains (pointed by arrow head) **E** sap-feeding larva, dorsal **F** spinning larva, dorsal view **G** leaf with a single *P. furcata* mine on adaxial surface (numbers indicate position of histological sections presented in Fig. 8; open and closed arrows indicated respectively the beginning and ending of the mine) **H** pupal cocoon, latero-dorsal **I** pupa, dorsal **J** pupal exuvium protruded from cocoon after adult emergence (close arrows points to pupal exuvium). Scale bars: 0.25 mm (**C**), 0.5 mm (**D**), 2 mm (**E, F**), 20 mm (G), 2.5 mm (**H–J**).

Figs 1 , 2 , 3 , 4 , 5 , 6 , 7

#### Type locality.

Peru, Arequipa, Characato [16°27'S, 71°28'W], 2400 m.

#### Specimens examined.

***Holotype***: Peru • ♂; Arequipa, Characato; 16°27'S, 71°28'W; 2400 m a.s.l.; VIII–IX.2018; J. Cerdeña, H. Vargas & J. Farfan leg.; reared from pupae collected on *Baccharis alnifolia* (Asteraceae); MUSM. ***Paratypes***: same data as for holotype • 1 ♂, 1 ♀; MUSM; • 1 ♂, 1 ♀; MUSA_ENT 015142, 015143; • 2 ♂, 2 ♀; LMCI.

#### Other material.

Adults, pinned and dried, 5 ♂, 8 ♀, same data as for holotype, MUSA_ENT 015144, 015145, 015146, 015147, 015148, 015149, 015150, 015151, 015152, 015153, 015154, 015155, 015156. Genitalia preparations (MUSA_Gent_015142, 015143, 015146, 015147, 015148), mounted on slides, with the same collection data. Immature stages (11 sap-feeding larvae, 06 spinning larvae, 20 pupae) preserved in 70% ethanol, with the same collection data, but with dates VIII–IX.2018, IV.2019 or XI.2019, MUSA.

#### Diagnosis.

Adults of *P. furcata* can be distinguished from all other known species of Neotropical *Phyllocnistis* in the forewing pattern by a combination of the following characters: ground color silver, four distinct transverse fasciae; transverse fasciae 1 reduced to the costal margin and mesally fused to longitudinal fascia, both not connected to transverse fascia 2; transverse fascia 3 almost reaching the middle portion of the wing. In the male abdomen, by the presence of two pairs of coremata on abdominal segment VIII, one pair consisting of wide rounded flat scales, a character not found in other Neotropical *Phyllocnistis*. In the female genitalia, by presenting a remarkable forked-shaped signum with four elongated spines on the distal margin. This species is similar to *P. wygodzinskyi* Hering, 1958 and *P. sexangula* Meyrick, 1915, in having similar patterns of fasciae. However, *P. wygodzinskyi* has a large black blotch at the inner border of the longitudinal fascia, and *P. sexangula* presents a small blotch close to the inner border of the longitudinal fascia, while *P. furcata* has no additional mark on the forewing.

#### Adult.

(Figs 1, 2). **Description. Male**: Forewing length 3.10–3.33 mm (*N* = 5). Head: Vestiture silvery pale brown, completely covered with smooth, broad, scales slightly overlapping anterior margin of eyes (Fig. 1B). Antennae light brown dorsally becoming dark towards apex and silvery white ventrally, approximately equal to length of forewing (Fig. 1A, F). Labial palpus slender, ~ 0.4 mm in length, covered with light grey scales (Fig. 1F). Proboscis without scales, slightly longer than labial palpus (Fig. 1F). Thorax: Forewing ground color silvery white; with light orange longitudinal (lf) and transverse (tf) fasciae (Fig. 1A, E); if bordered by dark brown scales, extending 2/3 length of wing from base of costa, and connected with tf1 apically; tf1 not reach the inner margin, restricted between the costal margin and lf; tf2 separate from tf1, lightly convex, crossing the wing entirely; tf3 separate from tf2, but not reach the inner margin; tf4 separate from tf3, crossing the wing entirely. Apex of forewing with a well-marked black spot. Costal strigulae, light orange, emerge from the base of transverse fasciae. Apical strigulae, dark brown, emerge from black spot. Inner marginal fringe varies from orange to dark brown. Hindwings light pale brown gray, with long light brown fringes. Legs light gray except dark brown over dorsal surface of femur, tibia, and tarsus of foreleg. Abdomen length ~2.0 mm, dark grey covered with silvery pale brown scales, two pairs of coremata present laterally on segment VIII (Fig. 2A), one pair consisting of a set of flat and long scales and the other pair consisting of wide rounded flat scales (Fig. 2D). Whether the wide rounded flat scales function as coremata by themselves or appendages of the long ones remains unknown.

***Male genitalia***: Uncus absent. Tegumen membranous, approximately equal to length of the valva, with spines arranged laterally from the base to the medial region. Saccus V-shaped, well developed, ~0.8 × the size of valva. Valvae digitiform and slightly convergent from the base to the apex, apex with small spine, setae randomly arranged along the valva getting shorter in the distal part (Fig. 2C). Phallus slender and with a slightly convex apex, weakly sclerotized, wrinkled cylinder, lightly longer than valva; cornuti absent (Fig. 2B).

**Female**: Forewing length 3.30–3.41 mm (*N* = 5). Color and pattern very similar to that of male, but head vestiture with light silvery scales (Fig. 1D). Hindwings light silvery gray with long silvery fringes and abdomen color light brown covered with silvery scales (Fig. 1C). VII abdominal sternum trapezoid, anterior margin thickened.

***Female genitalia***: Papillae anales slightly sclerotized, covered with hair-like setae. Posterior apophyses ~2.4 × length of anterior apophyses (Fig. 2F). Ostium bursae posterior to sternum 7. Ductus bursae completely membranous, slender, elongate, over 6.0× length of posterior apophyses (Fig. 2E). Corpus bursae slightly elongated, ~0.3 × length of ductus bursae, mainly membranous with three signa; a prominent fork-shaped signum on basis, resembling a garden fork, that occupies ~0.5 × length of corpus bursae with four elongated spines distally projected, and two small signa irregular in shape with minute dark spots on distal portion and also scattered sclerotized pellets on the bursa wall (Fig. 2G). Ductus seminalis membranous, narrow, inserted in base of corpus bursae.

#### Immature stages.

The number of larval instars was not determined, with three sap-feeding instars suspected and one spinning instar.

#### Egg.

(Fig. 7C). Flat, slightly ellipsoid; ~0.4 × 0.25 mm; chorion translucent; aeropyles, micropyles, and external ornamentation not observed.

#### Sap-feeding larva.

(Figs 3A, 4, 7E). Body flattened dorsoventrally, yellowish translucent (Fig. 7E). Length of largest larva examined ~ 4.5 mm. Head brown, prognathous, setae absent (Fig. 4A–C). Two pairs of small stemmata located in the lateral region (Fig. 4F). Antenna 3-segmented, with four sensilla, two stout ones located on the second segment and two on the distal segment, one spiniform and other stout (Fig. 4F). Labrum slightly bilobed with small epipharyngeal spines, which are of greater size in the lateral region (Fig. 4D). Labium slightly bilobed with small spines near distal margin (Fig. 4E). Spinneret present, in the form of a transverse slit. Maxillary and labial palpi absent. Legs and prolegs absent (Fig. 3A). Thorax with prothoracic light-brown dorsal shield in the form of a trapezoid (Figs 3A, 4G). Thoracic and abdominal segments without setae. Circular spiracle laterally on segments T1 and A1–A8 (Fig. 4H). Caudal abdominal segment slightly bilobed distally (Fig. 4I).

#### Spinning larva.

(Figs 3B, 5, 7F). Body yellowish, cylindrical, wider along the thorax and first abdominal segments, narrowing towards the posterior region, covered with microtrichia (Figs 3B, 7F). Approx. 5.00 mm maximum length. Head capsule weakly sclerotized, with anteriorly pronounced trophic lobe (Figs 3B, 5A–C). Stemmata absent. Antenna short, three-segmented, with five sensilla (Fig. 5F). Clypeal region with three pairs of setae (Fig. 5D). Maxillary palpi, represented by a pair of short sensilla. Spinneret short (Fig. 5E). Thorax with slightly pronounced prothoracic dorsal shield (Fig. 3B). Legs and prolegs absent. A single ambulatory callus ventrally on center of meso- and metathorax (Figs 3B, 5G). One pair of smaller ambulatory calli ventrally on A3–A7 (Figs 3B, 5I, J). One pair of lateral campaniform sensilla on A2–A9 (Fig. 5K). Caudal abdominal segment slightly bilobed distally (Fig. 5L).

#### Pupa.

(Figs 3C, 6, 7I). 1 Coloration changing from light yellowish during early stage of pupation to yellowish brown later in development (Fig. 7I). Approx. 5.00 mm maximum length. Cocoon-cutter triangular, concave dorsally (Fig. 6A–C) with serrated lateral edges (Fig. 6D). Frons with two pairs of large frontal setae close labrum (Fig. 6E). Labrum ellipsoidal (Fig. 6B). Antenna long and straight, extending to abdominal segment A7; forewing extending to A6 (Fig. 3C); prothoracic, mesothoracic and metathoracic legs reaching segments A3, A5 and A8, respectively (Fig. 3C). A pair of long setae, latero-dorsally on meso-, metathorax and A2 (Figs 3C, 6F). Lateral setae on abdominal segments A3–A7 (Fig. 3C); those of meso-, metathorax, A2–5 with dentate apex (Fig. 6I), those of A6–7 with clavate apex (Fig. 6H). A8 segment with a pair of acute setae latero-dorsally directed posteriorly (Fig. 6K). One pair of conspicuous spiracles up to A2–A7 (Fig. 6J). Dorsum of A1–A7 with a pair of curved, large spines, projecting laterally, from A2 to A7 with a variable sized patch of smaller spines projecting posteriorly between them (Fig. 6G). One pair of small lateral spines on the pleural region from A1 to A7 (Fig. 6H). Pleural region of body and last four abdominal segments covered by microtrichia (Fig. 6K, L). A pair of slightly divergent acute processes from caudal apex on last abdominal segment (Fig. 6K, L).

#### Etymology.

The species name *furcata*, from the Latin adjective *furcatus*, *furca* meaning fork, alludes to the large and prominent form of the signum present in the female genitalia, resembling a garden fork.

#### Host plant.

(Fig. 7B). *Baccharis alnifolia* Meyen & Walp. (Asteraceae) is the only host plant known for the immature stages of *P. furcata*. This species is distributed from Peru to northern Chile, with an altitudinal range between 2400–3800 m (Beltran et al. 2006; Rodriguez et al. 2018). In Peru, *B. alnifolia* inhabits the western slopes of the Andes, distributed from the departments of La Libertad to Tacna (Beltran et al. 2006). It is commonly known as “chilca”, a shrub that reaches a height of 1.5 to 3 meters, and grows predominately on river banks (Brako and Zarucchi 1993).

#### Distribution.

*Phyllocnistis furcata* is known only from the type locality, Characato, Arequipa, Peru (Fig. 7A).

#### Life history.

**Figure 8.**
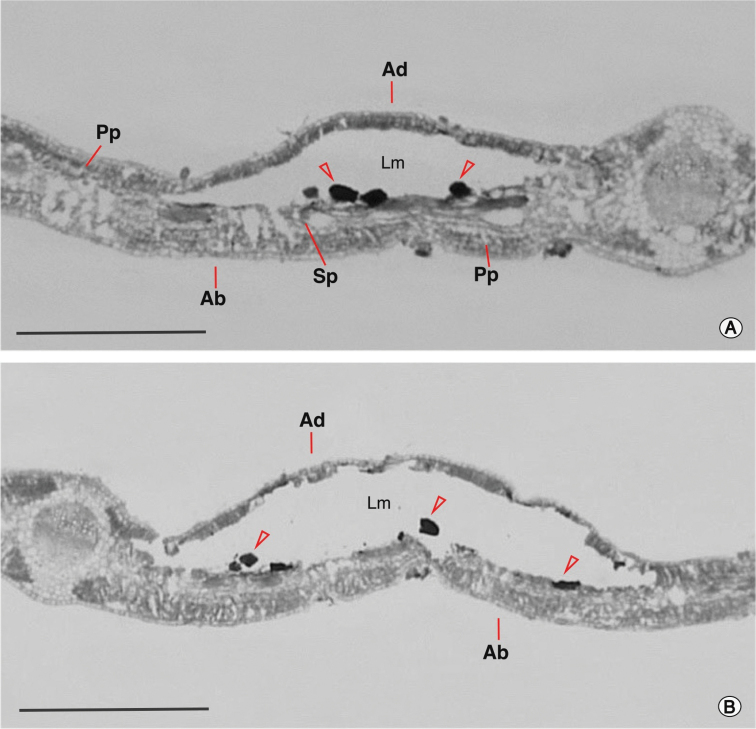
Transverse histological sections of *P. furcata* sp. nov. mine on *Baccharis alnifolia* Meyen & Walp. (Asteraceae) leaf **A** intermediate portion (location indicated by line 1 in Fig. 7G) **B** final portion (location indicated by line 2 in Fig. 7G). Feces are indicated by open arrows. Ab, epidermis of abaxial surface; Ad, epidermis of adaxial surface; Lm, leaf mine; Pp, palisade parenchyma; Sp, spongy parenchyma. Scale bars: 0.5 mm.

(Figs 7C–J, 8). *Phyllocnistis furcata* mines are serpentine throughout their length, initially narrow, increasing in width to the end of the mine, covering most of the area of the leaf (Fig. 7G). Mines were found either on young leaves under development or fully expanded ones, and almost all began near the midrib and extended along it. We found the majority of mines on the abaxial side of the leaf, and fewer on the adaxial side of the leaf. Most mines were found singly on a leaf; however, sometimes mined leaves carried two mines, either two on the abaxial side or one on each side. Mature mines are light green in color (Fig. 7G). Larvae are sap-feeders during the first instars (Fig. 7E) and are specialized in the palisade parenchyma, leaving the epidermis layers and generally the spongy parenchyma intact (Fig. 8). During the last spinning instar, it does not feed, but spins a cocoon within which pupation occurs (Fig. 7F). The cocoon is endophyllous, located on the final portion of the mine, during construction leading to a fold outside the leaf typical for *Phyllocnistis* (Fig. 7H). Before adult emergence, the anterior half of the pupa (head and thorax) protrudes out, while the posterior half remains in the pupal cocoon (Fig. 7J). In the examined mines, ~20% had a living and not parasitized larva or pupa. The remaining mines (~80%) were either empty or contained larvae or pupae which were either dead or parasitized by unidentified species of Hymenoptera; the affected stages varied from early sap-feeding larvae to pupal stage. Our field collection data indicate that the species may occur all year around in the area, with higher densities found in April and November.

#### Molecular data.

**Figure 9.**
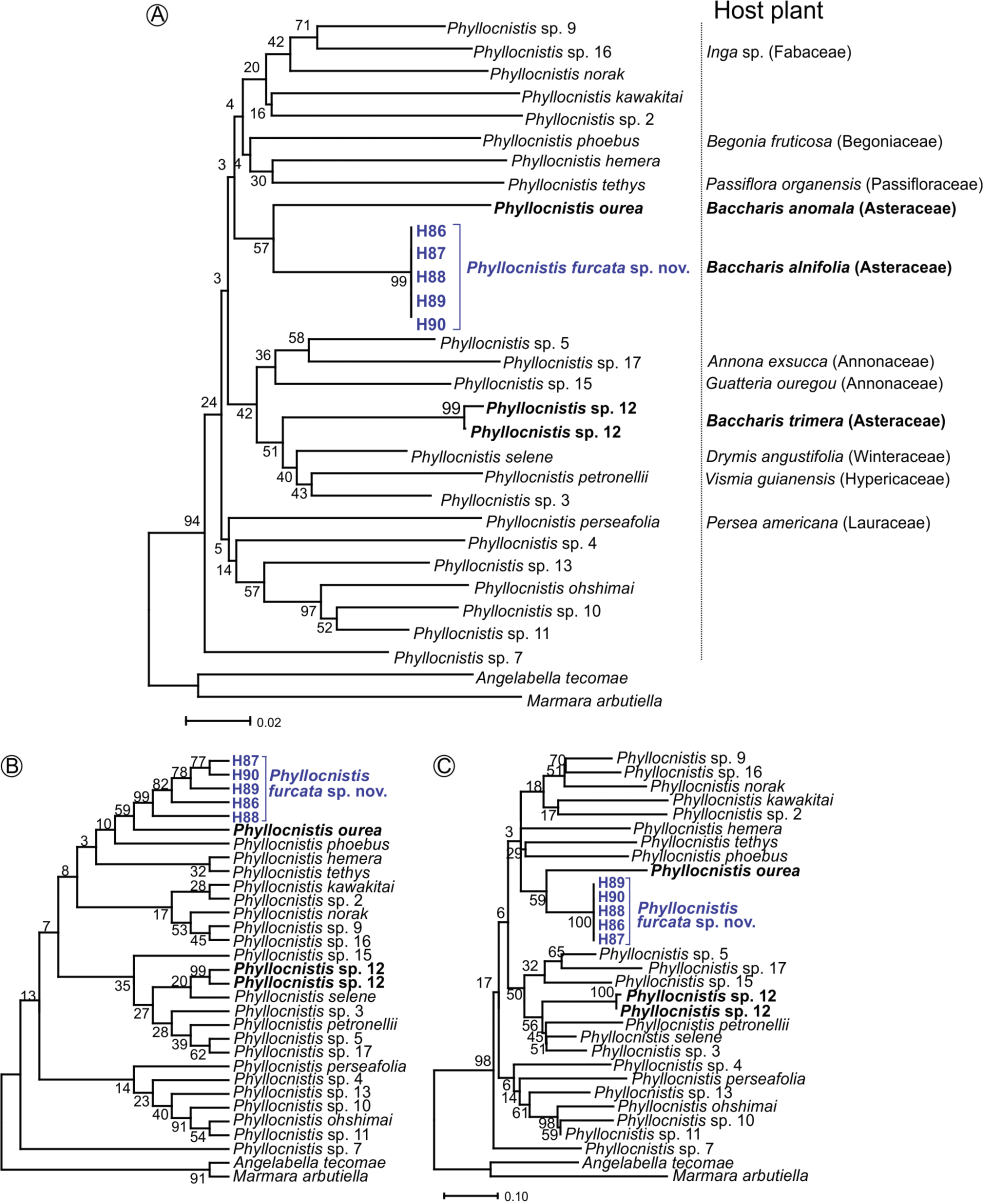
COI trees showing the specific classification of *Phyllocnistis furcata* sp. nov. (blue), and its position among 23 Neotropical *Phyllocnistis* lineages **A** phylogeny inferred using the Neighbor-Joining method with Kimura 2-parameter model. Host plants, when known, are indicated for each species [data were obtained from Brito et al. (2017) and BOLD database] **B** maximum parsimony consensus tree (length 1006, consistency index 0.3315, and retention index 0.4675) **C** maximum likelihood tree using the general time reversible model of sequence ecolution. –ln likelihood = 4913.93 The percentage of replicate trees in which the associated taxa clustered together in the bootstrap test (1000 replicates) are shown above the branches in A, B, and C. *Angelabella tecomae* Vargas & Parra, 2005 (Oecophyllembiinae) and *Marmara arbutiella* Busck, [1904] (Marmarinae) were used as outgroups. Bold indicates species/lineages that use *Baccharis* species as host plants.

(Fig. 9). The five DNA barcodes obtained for *P. furcata* (intraspecific distance = 0%) fall within the same clade, supporting the identification of the new species (Fig. 9). The nearest neighbor (BS = 57) is *P. ourea* (Fig. 9A), a *Baccharis*-feeding species. This pattern is consistent in MP and ML analysis (Fig. 9B, C, respectively), with node support (BS = 59). The mean distance between *P. furcata* and Neotropical *Phyllocnistis* (14.8%) is near the overall divergence within the genus (15.3%) and Neotropical groups (15.3%) (Table 2). The lowest divergence was observed between *P. furcata* and *P. ourea*. However, the *Baccharis*-feeding lineage *Phyllocnistis* sp. 12 showed high divergence distance (14.7%), similar to other species from the Neotropics.

**Table 2.** Mean genetic distance (minimum – maximum) based on COI sequences using Kimura-2 parameters method for distinct phylogenetic arrangement of *Phyllocnistis* species, with special reference to *Phyllocnistis furcata* sp. nov. N, number of specimens used in the dataset.

Group	N	Mean (minimum – maximum)
All named species of *Phyllocnistis*	35	15.4% (3.3–22.1)
Neotropical *Phyllocnistis* (described + undescribed species)	29	15.3% (8.9–21.6)
*P. furcata* vs Neotropical *Phyllocnistis*	29	14.8% (11.9–18.5)
*P. furcata* vs. *P. ourea*	6	11.9%
*P. furcata* vs. *Phyllocnistis* sp. 12	7	14.7%

## Discussion

*Phyllocnistis* is one of the most species-rich genera of gracillariids, in which a number of taxa were recently described in the Neotropical region, predominantly from tropical and subtropical forests (Davis and Wagner 2011; Brito et al. 2012, 2016, 2017; Lees et al. 2013). Herein we describe an Andean new species, *P. furcata*, based on morphological and molecular characters that clearly separate it from congeneric species. The COI tree showed a monophyletic status for the new species, and different methods of reconstruction support the inference of its sister relationship with *P. ourea* among Neotropical lineages.

Overall, adults of *P. furcata* resemble the majority of Neotropical *Phyllocnistis* in general aspects of forewing pattern (Brito et al. 2017, 2019; Fochezato et al. 2018); nevertheless, comparing *P. furcata* with *P. ourea* and *P. baccharidis*, all associated with *Baccharis* as host plants, there are no characters in the wing pattern that group them together; however, they share the presence of a small stout spine at the apex of the valva in the male genitalia. However, a high genetic distance (11.4%) is found between *P. furcata* and *P. ourea*, suggesting either an ancient divergence of these sister species or incomplete sampling of Neotropical species, taking into account that much more *Phyllocnistis* species could be expected associated with the large genus *Baccharis* (440 sp), and what could also be expected the close relationships among these micromoths.

*Phyllocnistis ourea* clustered in all COI analysis as the closest related to *P. furcata*, sharing the same genus of host plant (*Baccharis anomala*), which could indicate a clade which is associated only with *Baccharis*. However, the undescribed *Phyllocnistis* sp. 12 (Brito et al. 2017), which feeds on *Baccharis trimera*, did not cluster in that group. Moreover, this lineage presents a high genetic divergence (ca. 15%) from *P. furcata*, near to the mean divergence found of all *Phyllocnistis* species, suggesting a convergent evolution of host plant use. Such pattern is distinct to described for a species group of *Phyllocnistis* that feed on Salicaceae, recovered as a single evolutionary clade in a COI tree (Kirichenko et al. 2018). Further inclusion of *P. baccharidis*, and eventually more species/lineages (currently unknown) associated with *Baccharis*, together with a multi-locus approach, will allow one to make more robust phylogenetic inferences and shed light on the diversification of Neotropical *Phyllocnistis* related to its host plants.

Interestingly, males of *P. furcata* possess two pairs of coremata on abdominal segment VIII. Only one pair, formed by long, slender, flattened scales is found generally in species of *Phyllocnistis* in which the coremata are described (e.g., Kawahara et al. 2009; Brito et al. 2012, 2019; Kirichenko et al. 2018). *Phyllocnistis furcata* presents one pair similar to those in other congeneric species, and a second pair formed by wide rounded flat scales, which may represent the first report of this type of structure for the genus. Furthermore, another interesting aspect is observed in females of *P. furcata*, where one of the three signa of the corpus bursae is prominent (~0.5 × length of corpus bursae) with a particular fork-shaped appearance not observed in any other species of *Phyllocnistis*. This partially resembles the only signum of *P. tropaeolicola* Kawahara, Nishida & Davis, 2009, which has the form of a narrow band with two spines projecting inwards (Kawahara et al. 2009), but the spines are more prominent in *P. furcata*. *Phyllocnistis drimiphaga* Kawahara, Nishida & Davis, 2009 is another species with one of its two signa of large size, but five short spines arise from this signum. The remaining species of this genus mostly contain a small pair of fusiform signa or a single signum that occupies less than 2/3 of corpus bursae (Kirichenko et al. 2018; De Prins et al. 2019). Also, there is no information about the variability of coloration pattern between females and males in other species of *Phyllocnistis*, notably that males are slightly darker than females in *P. furcata*. This has not been observed in other species of the genus, and should be reviewed in detail in the future.

The knowledge of immature stages of *Phyllocnistis* is also insufficient. In the majority of the species of the genus whose pupal morphology is described, pupae are characterized by a well-developed cocoon cutter and some abdominal terga (generally A2–A7) with a pair of prominent laterally curved spines and many small spines medially (Kobayashi and Hirowatari 2011; Brito et al. 2012, 2019; Fochezato et al. 2018; Kirichenko et al. 2018; Liu et al. 2018). However, a few species do not match this pattern perfectly. For instance, *P. subpersea* Davis & Wagner, 2011 has the cocoon cutter in the form of a pair of stout conical processes with a strongly recurved subapical spine (Davis and Wagner 2011). Curved spines of the abdominal terga are absent in two Neotropical species (Brito et al. 2012, 2019) and are present only on two segments of *P. citrella* (Kobayashi et al. 2013). The pupa of *P. furcata* matches well the general pattern of the genus. In addition, the prominent curved spines are present on A1–A7, a condition also found in *P. ourea*, whose larvae also feed on *Baccharis* (Brito et al. 2019). The apex of the lateral setae of A6 and A7 is clavate in the pupae of these two *Baccharis*-feeding *Phyllocnistis*. However, this condition is also found in other species whose larvae are associated with other plant families (Kawahara et al. 2009; Davis and Wagner 2011; Brito et al. 2019). In the spinning larva, a single ambulatory callus placed ventrally at the center of the meso- and metathorax is found in *P. ourea* (Brito et al. 2019), *P. furcata*, and also in the Thymelaeaceae-feeding *P. hemera* Brito & Fochezato, 2018, while lateral sensilla on abdominal segments are found in *P. hemera* (Fochezato et al. 2018) and *P. furcata*. In the sap-feeding larva, the presence of two stemmata, like in *P. furcata*, has been described for *P. ourea* and the Winteraceae-feeding *P. selene* Brito & Moreira, 2017 (Brito et al. 2019). Certainly, the external morphology of the immature stages should be explored in additional species of *Phyllocnistis* to have a more realistic perspective of the actual variation and its relationship with ecology and evolution.

The few studies in which the damage pattern caused by leaf miner larvae of Gracillariidae has been characterized using histological sections, suggest that its feeding activity can either be restricted to specific tissues throughout the leaf miner stage or the consumed tissues can change with larval ontogeny (Brito et al. 2012; Body et al. 2015; Moreira et al. 2018; Pereira et al. 2019; Vargas-Ortiz et al. 2019). In the case of *Phyllocnistis*, the larvae of *P. citrella* feed only in the epidermis of *Citrus* (Rutaceae) (Achor et al. 1997), and those of *P. tethys* Moreira & Vargas, 2012 on the spongy parenchyma of *Passiflora organensis* Gardn. (Passifloraceae) (Brito et al. 2012). Furthermore, larvae of *P. hemera* feed initially on the epidermis and later on the palisade parenchyma of *Daphnopsis fasciculata* (Meisn) Neveling (Thymelaeaceae) (Fochezato et al. 2018). The feeding behavior of larvae of *P. furcata* was found to be restricted to the palisade parenchyma of *B. alnifolia*.

The mines of *P. furcata* are found on adaxial and abaxial surfaces of the leaf. In contrast, those of *P. ourea*, another *Baccharis*-feeding *Phyllocnistis*, are restricted to the adaxial surface of the leaf of its host *B. anomala* (Brito et al. 2019). Despite histological descriptions of leaves of *B. anomala* mined by *P. ourea* not being available, it appears that the remarkable difference between the distribution pattern of the mines of the two species in the leaves of their hosts could be due, at least in part, to differences in leaf anatomy of the two hosts and the ability of the larvae to feed on palisade parenchyma. The organization of the mesophyll in *B. alnifolia* is isobilateral, with two or three layers of palisade parenchyma in each side and one or two layers of spongy parenchyma in the middle, a pattern reported for several species of *Baccharis* (Budel et al. 2018; Ornellas et al. 2019), while the organization of the mesophyll of *B. anomala* is dorsoventral, with two or three layers of palisade parenchyma and approximately three layers of spongy parenchyma (Budel and Duarte 2008). Given this diversity in leaf tissue structure, additional studies are needed to better understand the ecology and evolution of herbivory in *Phyllocnistis*. Certainly, histological descriptions of leaves mined by additional species of this genus will be helpful to propose hypotheses.

The known distribution of endemic species of *Phyllocnistis* in the western slopes of Peruvian Andes was previously restricted to the type localities of the two species described at the beginning of the last century by Edward Meyrick (1915, 1921) around the Department of Lima, in central Peru. Thus, the discovery of *P. furcata* in the Arequipa Department, as a result of recent evaluations of the Microlepidoptera fauna, provides the first record of this genus from southwestern Peru, at 2400 m, where the vegetation is dominated by xeric shrublands with abundant cacti (Montesinos et al. 2012, Heim 2014). However, as already mentioned, the taxonomic diversity of gracillariid species remain poorly studied in Peru, due to the absence of local specialists and collections of micromoths in general. As discussed in Brito et al. (2016), there exists a “taxonomic impediment” for the progress of studies on Neotropical gracillariids in general. Therefore, regional revisions of micromoth faunas would represent an important advance to the knowledge of this diverse group in Peru (e.g., Davis et al. 2020), particularly in areas that are subject to the highest rates of anthropic environmental degradation, like the environments of the southern Andes of Peru.

## Supplementary Material

XML Treatment for Phyllocnistis furcata
